# Rationally Modified Estrogen Receptor Protein as a Bio-Recognition Element for the Detection of EDC Pollutants: Strategies and Opportunities

**DOI:** 10.3390/ijerph120302612

**Published:** 2015-02-27

**Authors:** Mattia Pedotti, Valentina Elisabetta Viviana Ferrero, Teresa Lettieri, Pascal Colpo, Stephane Follonier, Luigi Calzolai, Luca Varani

**Affiliations:** 1 Institute for Research in Biomedicine, Universita’ della Svizzera italiana (USI); via Vela 6, 6500 Bellinzona, Switzerland; E-Mail: mattia.pedotti@irb.usi.ch; 2 European Commission—Directorate General—Joint Research Centre, Institute for Environment and Sustainability, Ispra, Varese 21027, Italy; E-Mails: valentina.ferrero@jrc.ec.europa.eu (V.E.V.F.); Teresa.lettieri@jrc.ec.europa.eu (T.L.); 3 European Commission–Directorate General–Joint Research Centre, Institute for Health and Consumer Protection, Ispra, Varese 21027, Italy; E-Mails: pascal.cplpo@ec.europa.eu (P.C.); luigi.calzolai@jrc.ec.europa.eu (L.C.); 4 CSEM Landquart, Bahnhofstrasse 1, Landquart 7302, Switzerland; E-Mail: stephane.follonier@csem.ch

**Keywords:** estrogen receptor, EDC, biosensor, pollutants

## Abstract

The estrogen receptor protein (ER) can bind a vast number of organic pollutants widely spread in the environment and collectively known as Endocrine Disrupting Chemicals, EDCs. Its broad selectivity makes it an ideal bio-recognition element for the detection of EDCs. Here we describe the strategy and rationale for the design of ER based biosensors and assays that generate a signal in the presence of EDCs. The opportunity to use either natural or rationally modified ER molecules is discussed. The latter approach was successfully applied in the EU-FP7 project RADAR, with the aim to develop a novel biosensor for the detection of organic pollutants both in the environment and in commercial water products.

## 1. Introduction

Endocrine Disrupting Chemicals (EDCs) are a class of organic pollutants with an ever increasing presence in the environment. Sources of EDCs are ubiquitous in both industrial and consumer context, including industrial and residential (plastic products, pharmaceuticals, detergents) as well as agricultural waste. There is an increased demand for the detection and monitoring of these pollutants in the environment both at the consumer and legislative level. Hundreds of new EDCs with little or no toxicological characterization are released each year. Furthermore, other molecules that are not toxic per se can be degraded in the environment and transformed into EDC pollutants. 

Given the sheer number of existing, new and unknown EDCs, designing chemical assays or detection strategies for each individual compound is not obvious. Studying the biological action of EDC compounds, however, offers a chance to develop assays and sensors capable of detecting the presence even of unknown, potentially toxic chemicals.

Vertebrates utilize the estrogen receptor signaling pathway to regulate gene expression in the presence of steroidal hormones. This pathway plays a key role in development and maintenance of normal sexual and reproductive functions and has also effect on the cardiovascular, musculoskeletal, immune and central nervous systems [1].

**Figure 1:**
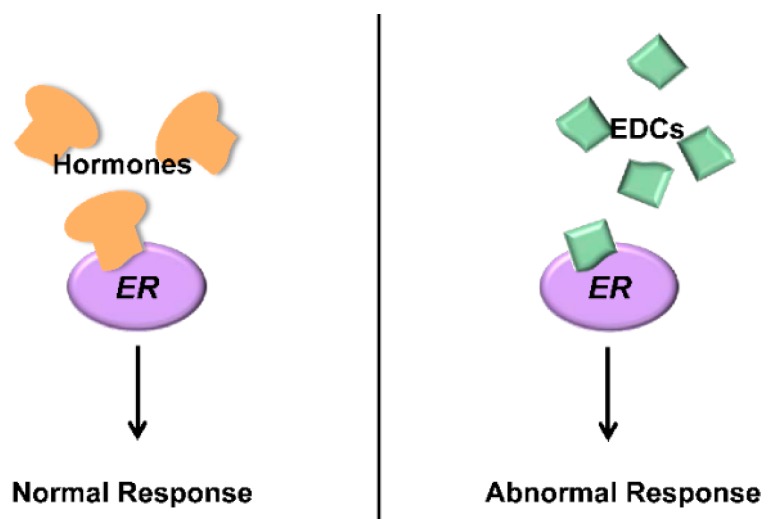
Estrogen Receptor protein (ER) regulate gene expression after binding hormones, schematically shown on the left. Endocrine Disrupting Chemicals (right) bind ER and provoke an abnormal physiological response, either inhibiting or untimely activating the normal ER functions. Many diseases are associated with Endocrine Disrupting Chemical (EDCs).

Estrogen Receptor (ER) is a protein that, in the presence of hormones, can regulate the above mentioned signaling pathways. During a normal response, 17β-estradiol binds to ER and activates the signal transduction resulting in regulation of gene expression [2]. Besides its natural ligands, ER can bind several other chemical compounds [3,4]; once bound, these can inhibit or abnormally stimulate the ER activity, disrupting its effects (Figure 1). Every molecule that can bind to ER and interfere with its activity is defined as an Endocrine Disrupting Chemical (EDC). EDCs have been linked to several diseases such as cancer, malfunctions of the reproductive and immune system [5,6] and sex change in fish and amphibians [7,8,9,10,11,12,13]. 

Given the above, ER protein itself can be a tool for the detection of such organic pollutants. An assay or sensor device that can provide a signal if an organic molecule binds to ER can, in fact, be used to detect the presence of EDCs. No *a priori* knowledge on the compounds to be investigated is needed: any molecule that can bind to ER and give a signal in the assay or sensor has the potential to bind ER in the body, as well, exerting a toxic action.

**Figure 2:**
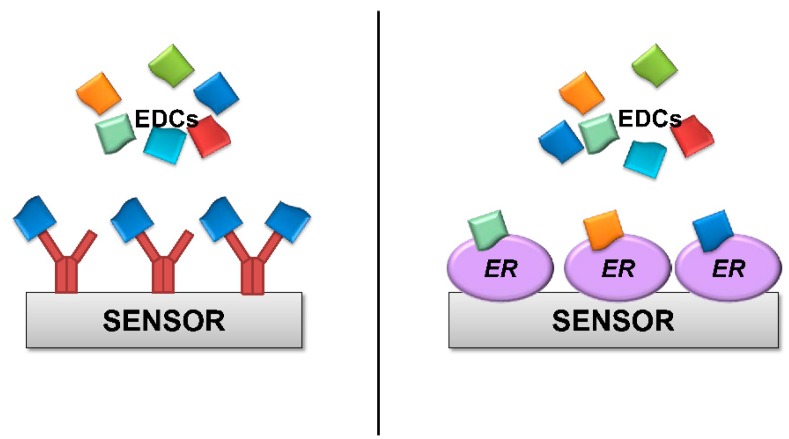
Schematic representation of a biosensor utilizing either antibodies (left) or ER protein (right) as bio-recognition elements. Antibodies, shown in red, are highly sensitive and usually highly specific. Each antibody would bind single EDCs and would not be able to detect the presence of others. By contrast, ER proteins bind any EDC. An ER based sensor would be able to detect the presence of any EDCs, even initially unknown compounds.

The concept of detecting a signal when a target binds to bio-recognition proteins has widespread uses with antibodies, either via biochemical (e.g., ELISA) or biophysical assays. However, using ER instead of antibodies as a bio-recognition element for EDCs has an important advantage. Antibodies are highly sensitive but are usually also highly specific (Figure 2); each antibody can only recognize a small number of similar molecules. Having different antibodies against the thousands of known EDC pollutants would be a daunting task, and it would be almost impossible to have antibodies against unknown EDC compounds. By contrast, any molecule that binds to ER protein is by definition a potentially toxic EDC. Detection of a signal in an ER based assay or sensor would thus alert to the presence of dangerous organic pollutants, even if these pollutants are initially unknown and not specifically searched for. The obvious disadvantage is that such assays would not be able to identify a specific pollutant; therefore, further analysis by traditional (and slower) methods would be required. However, alerting to the presence in the environment of any potential pollutant is certainly valuable. An ER-based sensor or assay would be used in routine controls and/or early warning systems. Detection of a potential threat by this method would then warrant further investigation.

## 2. Producing ER Protein as a Bio-Recognition Element

**Figure 3:**
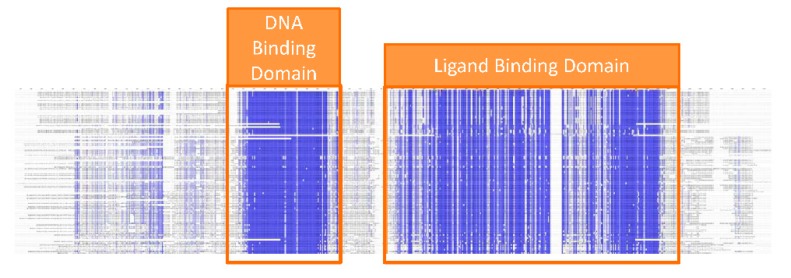
Sequence alignment of ER protein from different species. Blue color indicates conserved residues. The DNA binding and ligand binding domains are highlighted.

The Estrogen Receptor protein can be used as a bio-recognition element for the detection of organic pollutants. Given that all vertebrates have ERs, the first step for the design of a bio-sensor is choosing which species to derive the ER from. If the main focus of the bio-sensor is to detect compounds potentially toxic for humans, it would then be appropriate to use human ER as a bio-recognition element. Anything that binds to the biosensor, in fact, would also have the potential to bind to ER in the human body. Conversely, monitoring the presence of pollutants in water might suggest the use of ER from aquatic species. The choice is less critical than it might appear. Comparison of the protein sequence of ER from several species indicates that two regions of the protein are highly conserved: the DNA binding domain and the ligand binding domain [14,15,16] (Figure 3). 

The former domain is required to achieve regulation of gene expression whereas the latter is necessary and sufficient to bind estradiol and other ligands. Only the ligand binding domain, therefore, is strictly required as a bio-recognition element for EDCs. Production of the isolated ligand binding domain rather than the full ER protein may have practical advantages. For instance, smaller globular protein domains are often easier to produce in bacterial systems (see below) and are more stable over time. On the other hand, each protein is different and general rules are difficult to draw. Production of full, multi-domain proteins might be better suited for some application. In the EU-FP7 funded RADAR project we discovered that the isolated ER ligand binding domain is easy to produce, sufficiently stable over time and capable of detecting EDCs [17,18]. If the ER ligand binding domain is used as bio-recognition element, choosing which species to derive the protein from has very small impact: the domain is so highly conserved, especially the region responsible for binding EDCs (called ligand binding pocket), that any given protein sequence is suitable.

Having chosen the exact ER protein sequence to use, the subsequent step in the design of an ER based biosensor or assay is the actual production of sufficient quantities of the protein. “Sufficient” for laboratory testing usually means milligrams of purified and active protein. Industrial production would require apt scale up of the manufacturing protocols. Isolating and purifying ER protein from animals that naturally produce it would obviously be an impractical option. A better alternative is to produce recombinant ER protein from suitable expression systems, namely bacterial, eukaryotic or mammalian cell lines. The latter hosts yield proteins more similar to those present in animals. In particular, post-translational protein modifications such as glycosylation are not obtainable from production in standard bacterial systems. However, bacterial expression (typically in *E. coli*) is much cheaper, usually yields higher quantities of purified material and has less complicated regulatory requirements. For these reasons, bacterial systems are often preferred, provided that the produced protein is functional. Purification of the ER protein expressed in the system of choice is not particularly challenging and can be conducted according to standard techniques, often involving an affinity purification chromatography step followed by a size exclusion chromatography step [18].

## 3. Modify the Natural ER Protein

The ligand binding domain of the natural (wild type, wt) ER protein can be produced and used as a bio-recognition element capable of generating a signal if EDC pollutants are present in an analyzed sample. However, modification of the natural protein might be desirable for specific purposes. For instance, if ER needs to be linked to a solid support for an assay or biosensor, introduction of a chemical tag in the recombinant ER (e.g., an His-tag), might facilitate the task as well as aid in protein purification. In other cases the recombinant ER protein might not be stable for a sufficiently long amount of time. Improving shelf life can often be achieved by changing storage conditions (buffer, formulation, *etc*.). Other times, modification of the amino acid sequence of the protein might be necessary. Although a detailed explanation of this approach goes beyond the scope of this manuscript, one example might help to illustrate the process. Purified proteins tend to aggregate over time, leading to loss of activity. Aggregation is often initiated by the presence of hydrophobic residues (patches) on the protein surface, which attract similar patches on other molecules. Replacing these hydrophobic residues with hydrophilic amino acids in the recombinant protein has the potential to decrease aggregation and increase stability. 

Finally, the ER residues responsible for interaction with EDCs might be modified to increase the binding affinity, and thus improve sensitivity, for all or selected classes of pollutants. Achieving this result is not trivial but can be attempted with two general strategies. One option is to randomly mutate the ER protein sequence, generate as vast a library of different mutants as possible and then select the mutants with desired binding properties (e.g., higher affinity for a given chemical). The generation of random protein sequences can be achieved by synthetic gene libraries [19] or by yeast- or phage-display technologies [20,21,22]. 

**Figure 4:**
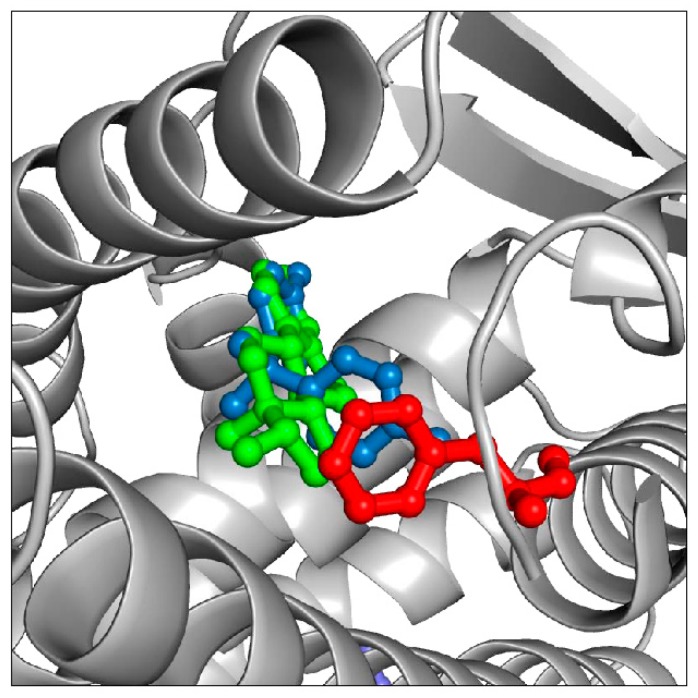
Part of the ER ligand binding domain is shown in grey cartoon, with the M421F mutation (see main text) shown in red. Computational simulations predict this ER mutant to have an increased affinity for estradiol (green) and bisphenol (blue). The two molecules cannot bind simultaneously but are shown superimposed in the ligand binding pocket for clarity.

A second approach involves the rational modification of specific residues that play important role in the interaction of ER with EDCs. These are best identified by structural analysis of the atomic, three-dimensional structure of ER protein in complex with various EDCs. Structural information allows, in fact, to visualize the chemical interactions responsible for EDC binding. Critical ER residues forming intermolecular contacts such as hydrogen bonds or aromatic interactions can be identified. Mutations in these positions can then be introduced to either disrupt intermolecular interactions and abrogate binding or, conversely, to favor binding of selected EDCs. Experimentally determined X-ray structures of ER protein bound to several different EDC compounds are available [4,23,24]. Binding of other EDCs of interest can also be simulated by computational docking algorithms [18]. The advantage is that computational simulations are usually fast and inexpensive. The disadvantage is that computational solutions often lack precision and accuracy. As an example, structural analysis based on both X-ray and computational data reveals that most EDCs bind to a hydrophobic binding pocket in the ER ligand binding domain. Since many EDCs have aromatic chemical moieties, increasing the aromatic content of the ER binding pocket might strengthen ER/EDC binding (and thus affinity) through the formation of energetically favorable intermolecular aromatic stacking interactions. Indeed, simulations suggest that mutating residue M421, in the ER ligand binding pocket, to F421 or Y421 might increase the affinity of ER to molecules such as estradiol and bisphenol (Figure 4). Furthermore, sequence analysis of ER proteins shows that residue M421 is mutated to F421 in some aquatic species. Since ERs with F421 exist in nature and are obviously stable and functional, it is likely that introducing a M421F mutation in any given ER sequence would not cause disruption of the ER structure and consequent loss of activity. Indeed, we were recently able to show that a M421F ER mutant can bind estradiol with approximately 10 fold higher affinity and bisphenol with 4 fold higher affinity [18], proving that the above described strategy is viable.

## 4. Conclusions

ER protein can be used as a bio-recognition element for the detection of a class of organic pollutants, collectively known as EDCs, that are toxic, widespread in the environment and whose monitoring and detection is increasingly required by legislation and consumer pressure alike. The advantages of using ER instead of antibody-based or chemical analysis is that any compound that exerts toxicity by binding to natural ER in animals will also bind to a recombinant ER protein in a biochemical assay or sensor. Even unknown and yet uncharacterized compounds can be detected, whereas they would fail to be recognized by antibodies or other specific assays. The main disadvantage of the broad selectivity linked to a recombinant ER bio-recognition element is that, although the presence of pollutants in a sample can be detected, such pollutants cannot be identified. Nonetheless, one can envisage the use of an early warning, ER based detection system that could signal the presence of potentially toxic EDCs. A positive sample would then be subjected to more expensive and time consuming chemical investigation with the purpose of identifying the organic pollutants.

In this manuscript we described the strategy and considerations that can lead to the design of ER based bio-recognition elements. These are applicable to other classes of natural protein receptors or enzyme capable of binding to, and thus detecting, different molecules. We recently used this strategy in the EU-FP7 research project RADAR, producing recombinant ER that could detect estradiol, an EDC, in a chromatography-mass spectroscopy assay more sensitive and convenient than currently available methods [17]. We were also able to generate rationally designed ER mutants with increased affinity for several EDCs [18] , leading to increased sensitivity in an ER based fluorescent assay. A label free, SPR based biophysical assay for the detection of selected EDCs has also been hinted to [25], although no finished biosensor has so far been produced.

The use of recombinant proteins as bio-recognition elements in biochemical and biophysical assays has steadily moved forward in recent years, becoming more affordable thanks to robotics, miniaturization and microfluidics. Recent advances in structural biology, computational simulations protein engineering allow us to move one step further: the natural proteins can be altered to ameliorate their properties. Protein mutations can positively affect the binding affinity for ligands (linked to assay sensitivity), selectivity and specificity or protein stability (particularly important for on-field biosensors). Mutating a protein to achieve a desired outcome remains a difficult endeavor; however, improved understanding of the principles of protein structure and protein interactions has greatly increased the success rate for protein engineering. Success is far from being guaranteed, but goals that were almost impossible a few years ago are nowadays worth pursuing.

Rational modification of natural protein receptors is an option certainly worth considering when planning the design of new bio-sensors.
